# How culture values shape leadership and employee well-being: insights from altruistic and egoistic perspectives

**DOI:** 10.3389/fpsyg.2025.1637594

**Published:** 2026-01-07

**Authors:** Zhirong Tian, Qian Wen, Haoming Ding, Yuan Zhu

**Affiliations:** 1 School of Economics and Management, Guangxi University of Science and Technology, Liuzhou, Guangxi, China; 2 Hoseo University, Asan-si, Republic of Korea; 3 School of Humanities, Arts and Design, Guangxi University of Science and Technology, Liuzhou, Guangxi, China

**Keywords:** leadership, altruistic values, egoistic values, employee happiness, affective organizational commitment, satisfaction with management

## Abstract

**Objective:**

Employee happiness is employees’ positive feelings about their work and quality of life. Previous research has mainly focused on employees’ own characteristics and behaviors, while there has been relatively little research on how leadership values affect employee happiness.

**Methods:**

A survey was conducted among employees in service industry companies in Guangdong Province, yielding 448 valid responses. Using empirical analysis, the study examines how leaders’ altruistic and egoistic values influence leadership effectiveness and, subsequently, employee happiness through emotional and cognitive mechanisms.

**Results:**

The findings show that leaders with altruistic values significantly improve leadership effectiveness, thereby improving employee happiness through a dual pathway: emotions cultivated by affective organizational commitment and cognitive appraisals as reflected in contact and satisfaction with management. These results highlight the key role of altruistic values in creating an organizational culture oriented toward employee happiness, underlining their importance in fostering trust, support, and collaboration within teams. In contrast, the study found that leaders with strong egoistic values had no significant positive impact on leadership, suggesting that overly self-centered values may undermine a leader’s credibility and influence among employees.

**Implications:**

This study provides a novel perspective on improving employee happiness by highlighting the importance of cultivating and promoting altruistic values in leadership development programs. It emphasizes the need for organizations to prioritize value-driven leadership practices that balance organizational goals and employee happiness, ultimately promoting a supportive and collaborative work environment.

## 1 Introduction

As workplaces pay more and more attention to satisfying employees’ sense of belonging and mental health, employee happiness has gradually become a core goal of organizational sustainable development (Rasool et al., 2021; Erkutlu and Chafra, 2016). An organization actively helps employees grow and promotes an environment that promotes personal achievement and overall satisfaction. Employees are facing increasing challenges due to increased workplace stress and the increasing overlap between work and personal life (Adisa et al., 2022). Strengthening the relationship between leaders and employees is essential to fully understand and improve happiness.

The challenges faced by employees have intensified significantly in recent years as workplace stress increases and the boundaries between work and personal life blur (Steffens et al., 2023). This challenge affects employees’ emotional stability, leading to burnout and reduced productivity. Therefore, the relationship between leaders and employees is particularly critical. Strengthening the quality of interaction between leaders and employees can help alleviate employees’ negative emotions caused by work pressure and provide employees with psychological support and development space (Wang C. et al., 2024). As an important measure of mental health, happiness is the core way for organizations to improve employee satisfaction, enhance team cohesion and stimulate innovation (Bani-Melhem et al., 2022; Salas-Vallina et al., 2017).

Happiness is an important measure of mental health (Burns and Crisp, 2022). Employee happiness in the workplace contributes to higher job satisfaction, greater emotional stability, and increased innovation (Algarni and Alemeri, 2023; Thompson and Bruk-Lee, 2021). Happiness enables employees to more actively cope with stress and challenges at work, stimulate employees’ intrinsic motivation, improve work efficiency and teamwork ability, and create value for the organization (Wulandari et al., 2024). In addition, Bellet et al. (2024) pointed out that positive employee happiness can also directly promote organizational performance and achieve higher sales. This shows that happiness is of great significance to employees’ personal development and overall organizational performance improvement. Previous literature has fully demonstrated that leadership behavior positively affects employee happiness. Romão et al. (2022) found that leadership coaching skills as a key factor actively promote employee happiness. Bani-Melhem et al. (2022) pointed out that excellent superior-subordinate relationships can enhance happiness through a service atmosphere, thereby further promoting employees’ innovative service behaviors. Therefore, the relationship between leadership and employee happiness reflects the direct impact of leadership behavior on employee mental health and reveals the key role of workplace atmosphere.

Although existing research has revealed the important impact of leadership on employee happiness (Romão et al., 2022; Bani-Melhem et al., 2022; Kaur and Kaur, 2024), the exploration of the antecedents of leadership is still insufficient. The role of personal traits that affect leader values in shaping leadership behavior has not yet been fully discovered. Values are the core driving force of personal beliefs and behaviors, reflecting the individual’s basic views and judgments on things (Gamage et al., 2021). They profoundly affect the leader’s interaction and decision-making style. Specifically, egoistic values focus on the leader’s own achievements, status, and pursuit of personal interests, while altruistic values focus more on the overall interests of the team and the needs of employees, and care and support promote team collaboration and employee growth. Clarifying the role of values in leadership, organizations can use values as key indicators in leader selection, training, and evaluation to ensure that leadership practices fit the organizational culture (Liao et al., 2024). They can also enhance employees’ happiness and sense of belonging by increasing leadership’s attention to employees’ psychological needs.

This study explores the antecedents that influence leadership and fills the gaps in the existing literature on the mechanisms of leadership formation and how personal leadership traits shape leadership. This study further reveals how leadership affects employee happiness through two paths: emotional level (affective organizational commitment) and rational level (satisfaction with management). The results of this study show that altruistic values are the main driving factor of leadership. Leaders with altruistic values tend to enhance employees’ sense of belonging and team cohesion by creating a united and collaborative work environment and atmosphere, thereby effectively improving the overall performance of employees. Sense of happiness. In contrast, the effect of egoistic values on leadership was not significant, suggesting that a lack of attention to employees’ psychological needs may limit the role of leaders in promoting employee happiness (Mireles-Hernández et al., 2024). At the emotional level, leaders make employees feel cared for and supported by enhancing their emotional attachment to the organization, thereby improving employees’ sense of belonging and psychological safety. This emotional bond helps relieve employees’ stress and anxiety, inspires employees’ loyalty and active commitment to the organization, and ultimately improves their happiness. At the rational level, leaders enhance employees’ trust and positive evaluation of the work environment by increasing employees’ recognition of management methods (He and Wei, 2022; Pincus, 2024). This sense of trust makes employees more willing to accept management policies, improves their overall satisfaction with the organization, and thereby improves work efficiency and happiness.

The rest of this study is structured as follows: Section 2 is a literature review and hypothesis, which proposes research hypotheses based on existing research; Section 3 is a methodology, which introduces data collection methods and variable measurement methods; Section 4 is a result analysis, which presents the results of the analysis model; Section 5 is a discussion and conclusion, which discusses the main findings and practical significance, and proposes the limitations of the study and future research directions.

## 2 Literature review and hypothesis development

### 2.1 The importance of leadership values

A leader’s personal values profoundly affect the quality of his or her relationships and interactions with followers (Kouzes and Posner, 2011), and also shape the leader’s behavioral patterns and ethical decisions in the organization (Russell, 2001). Values are the core driver of a leader’s behavior. They determine a leader’s focus on team and organizational goals, and influence how they shape organizational culture and respond to employee needs. Existing research has fully revealed the significant impact of leadership on employee happiness (Inceoglu et al., 2018; Abolnasser et al., 2023; Ilyas et al., 2023). Abolnasser et al. (2023) pointed out that transformational leadership can directly or indirectly improve employee happiness by shaping a positive organizational climate and enhancing employees’ psychological safety. Ilyas et al. (2023) further shows that people-centered ethical leadership helps build trusting relationships by reflecting the leader’s high ethical standards and concern for the well-being of employees, thereby improving employee happiness. Overall, these studies show that leadership plays a key role in improving employee happiness by promoting positivity in organizational culture, enhancing emotional communication, and enhancing employees’ sense of belonging.

Although existing research has made important progress in exploring the impact of leadership on employee happiness, research on the driving factors behind leadership is still insufficient. This study explores how leaders’ personal values affect their leadership behavior and employee happiness. From the perspective of values. Values serve as enduring principles that shape leaders’ decision-making orientations and ethical reasoning, influencing how they balance personal goals with collective interests. Drawing on Schwartz’s Value Theory (Schwartz, 1994; Schwartz, 1992), this study conceptualizes leadership values through two contrasting orientations: altruistic values and egoistic values. According to Schwartz (1992), values can be organized along a continuum of motivational goals, where altruistic values correspond to self-transcendence, prioritizing the welfare of others and collective harmony. While egoistic values correspond to self-enhancement, emphasizing personal success, power, and dominance.

Leaders’ core values determine how they deal with complex organizational environments, how they interact with employees, and their decision-making tendencies when facing ethical dilemmas (Eisenbeiss, 2012; Robinson et al., 2022). Leaders with altruistic values are more likely to adopt supportive and caring leadership behaviors, thereby playing a positive role in improving employee happiness. In contrast, leaders with egoistic values may be more concerned with personal interests and the short-term realization of organizational goals. Therefore, in the process of selecting and training leaders, organizations should pay more attention to the shaping and evaluation of leaders’ personal values (Alfalih and Ragmoun, 2025). Through value assessment, leaders with altruistic values can be selected. This helps optimize leaders’ behavior patterns and further promotes organizational performance by enhancing employees’ happiness and job satisfaction.

Moreover, recent evidence on ethical leadership (Mubarak et al., 2022b) suggests that leaders’ value orientations play a crucial role in shaping employees’ psychological states and work attitudes (Mubarak et al., 2021). This body of work strengthens our theoretical argument that leaders’ altruistic and egoistic values serve as foundational determinants of leadership effectiveness, which subsequently influence both the emotional and cognitive dimensions of employee happiness. By integrating insights from these studies, this research demonstrates how personal values at the leadership level activate dual mediating mechanisms to enhance employee well-being.

Taken together, building upon Schwartz’s theoretical framework, this study extends existing research by empirically examining how these two value orientations shape leadership effectiveness and employee happiness through both emotional and cognitive mechanisms. This theoretical integration moves beyond descriptive accounts of leadership behavior to uncover the underlying value systems that guide leaders’ interactions and their implications for employee outcomes.

### 2.2 The relationship between values and leadership

In social organizations, altruistic values prioritize the welfare and collective interests of others, emphasizing cooperation, support, and the achievement of shared goals (Ajmal et al., 2024). These values serve as a crucial foundation for fostering ethical behavior, employee care, and a strong sense of social responsibility, both in leadership practices and in broader corporate social responsibility initiatives (Amber et al., 2022; Ahmad et al., 2023). Leaders who embody altruistic values are more likely to prioritize the needs of their team members, balancing organizational objectives with a commitment to social responsibility. This approach strengthens trust and rapport among employees and enhances team cohesion and overall organizational performance (Kement et al., 2024; Ajmal et al., 2024). By promoting a supportive and inclusive environment, altruistic leaders play a pivotal role in creating a workplace culture that values collaboration, mutual respect, and ethical practices, ultimately contributing to sustainable organizational success (Hui and Wenan, 2022).

Egoistic values are rooted in individuals assessing actions primarily through the lens of personal interests, aiming to maximize outcomes by leveraging their own resources and capabilities (Gomes et al., 2023). Such a value orientation may undermine effective leadership because it tends to prioritize individual goals over collective needs and discourages shared decision-making and team cohesion. Previous research suggests that leaders driven by egoistic values often struggle to foster team-based collaborative decision-making due to their self-centered focus (Tan and Sousa, 2023; Ting and Ahn, 2023). Therefore, an egoistic orientation may compromise the leader’s ability to create a supportive climate and thus reduce perceived leadership effectiveness. Accordingly, we propose the hypothesis:

*H1*: Altruistic values are positively associated with enhanced perceived leadership.

*H2*: Egoistic values demonstrate a notable negative association with perceived leadership.

### 2.3 The relationship between leadership, affective organizational commitment, and satisfaction with management

Leadership is considered to be a key factor influencing employees’ affective organizational commitment (Pradhan and Pradhan, 2015). Affective organizational commitment refers to employees’ emotional attachment, identification, and sense of belonging to the organization (Allen and Meyer, 1990). Previous research has shown that leadership can significantly enhance employees’ affective organizational commitment through a variety of pathways. Transformational leadership enhances employees’ identification with organizational goals by clarifying expectations and providing personalized care, thereby enhancing affective commitment (Park et al., 2022). In addition, ethical leadership is also considered an important way to enhance affective organizational commitment by demonstrating fair and transparent behaviors (Al Halbusi et al., 2021).

Furthermore, Mubarak et al. (2022a) showed that employees’ psychological states—such as distress, resilience, and mindfulness—play a pivotal role in determining work outcomes, reinforcing the idea that leadership influences employees not only through observable behaviors but also through internal emotional processes. This aligns with our proposition that affective organizational commitment acts as an emotional pathway through which leadership values enhance employee happiness. Therefore, we hypothesize:

*H3*: The perceived leadership exerts a significant positive influence on affective organizational commitment.

Satisfaction with management is employees’ evaluation of leaders’ behavior, decision-making and management style, and is an important component of employees’ overall job satisfaction (Mohammad Mosadegh Rad and Hossein Yarmohammadian, 2006; Specchia et al., 2021). Previous research has shown that transformational leadership creates a supportive organizational environment, values employee needs, and actively listens to employees (Kim Quy et al., 2023). In addition, transformational leadership can enhance satisfaction by communicating with employees, clarifying development goals, empowering employees, and providing timely feedback (Tagscherer and Carbon, 2023).

Similarly, research has demonstrated that employees’ cognitive evaluations of their work environment significantly affect their well-being. Yasmin and Mubarak (2021) found that job satisfaction mediates the relationship between adverse workplace experiences and turnover intention, while Sarwar et al. (2021) showed that employees’ perceptions of organizational treatment shape their behavioral responses. These findings support our conceptualization of satisfaction with management as a key cognitive appraisal mechanism through which leadership values contribute to employees’ happiness. Therefore, we hypothesize:

*H4*: The perceived leadership has a significant positive effect on satisfaction with management.

### 2.4 The relationship between satisfaction with management, affective organizational commitment, and employee happiness

Employees’ personal feelings about their managers’ leadership style and decision-making play a key role in shaping their workplace experiences and overall job satisfaction (Abolnasser et al., 2023). Leadership behaviors that foster trust, fairness, and open communication positively influence employees’ perceptions of the workplace. Previous research has consistently demonstrated that higher levels of job satisfaction led to greater employee happiness, emphasizing the role of supportive and effective leadership (Abolnasser et al., 2023; Ju et al., 2024; Hafeez et al., 2024). In this study, high employee satisfaction, reflected in a sense of identification and belonging to the organization, is hypothesized to enhance overall happiness. This highlights the importance of leadership not only in achieving organizational goals but also in fostering a supportive environment that prioritizes employee happiness and positive workplace outcomes.

Affective organizational commitment refers to the emotional attachment of employees to their organization, encompassing a sense of identification and involvement (Mercurio, 2015). When employees establish a close emotional bond with the organization, they are usually more actively engaged in work and show higher work performance. Previous studies have consistently shown that affective organizational commitment positively impacts employee well-being by fostering a sense of belonging and purpose (Kustiawan et al., 2022; AlKahtani et al., 2021). In this study, employees with high emotional organizational commitment are hypothesized to approach work challenges with a more optimistic outlook and derive emotional satisfaction from their relationships within the team and organization. This highlights the dual role of emotional attachment in enhancing both individual and organizational outcomes. Therefore, we hypothesize:

**Figure 1:**
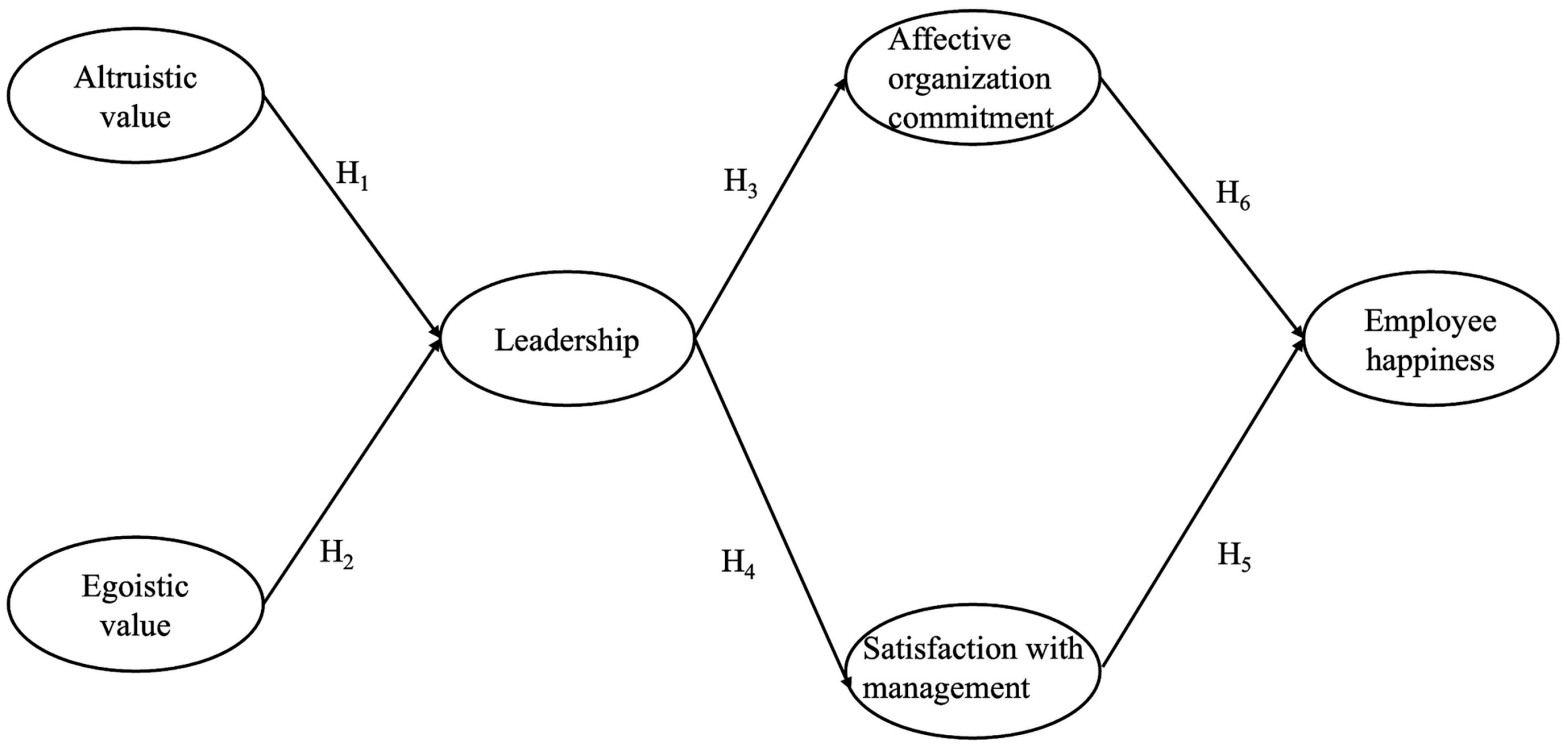
Research model.

*H5*: Satisfaction with management have a significant positive impact on employee happiness.

*H6*: Affective organizational commitment has a significant positive impact on employee happiness.

Based on the above hypotheses, a complete conceptual model for this study is developed. The model is presented in Figure 1.

## 3 Methodology

### 3.1 Questionnaire design

The design and modification of the questionnaire were based on existing validated measurement questions and were adjusted in accordance with the specific needs of the study. A 7-point scale was used, with options ranging from 1 (completely disagree) to 7 (completely agree), with 4 (neutral) in the middle. When designing the original questionnaire, the mature questionnaire content in related studies was referred to and translated from English into Chinese to ensure the accuracy and consistency of the language expression. To improve the applicability of the questionnaire among the target respondents, the research team conducted a pre-test and identified the areas of difficulty in understanding in the questionnaire through the feedback of the respondents. Based on the pre-test results, the expression and content of the questionnaire were modified several times to enhance the readability and fluency of the questionnaire and ensure that the measurement questions could reflect the information required by the research objectives.

The measurement of leader values is based on the relevant studies of Ahmad et al. (2024) and Prömpeler et al. (2023) and has been modified in combination with the context of this study. The measurement of leadership uses the scale of Rafferty and Griffin (2004), the measurement of Affective Organizational Commitment refers to Park et al. (2022), the measurement of Satisfaction with Management refers to Semedo et al. (2017), and the measurement of employee happiness is also based on the research of (Semedo et al., 2017). All specific measurement items and sources are shown in Table 1.

**Table 1:** Measurement items.

Construct	Code	Items	Source
Altruistic values	AV1	Equality: Advocating for equal opportunities and fair treatment for all individuals, regardless of differences.	Ahmad et al. (2024)
	AV2	A World in Peace: Striving for a harmonious society free from war and conflict.	
	AV3	Social Justice: Addressing and rectifying societal injustices while ensuring care and support for the vulnerable.	
Egoistic values	EV1	Social Power: Exercising influence and dominance over others to shape social dynamics.	Prömpeler et al. (2023)
	EV2	Wealth: Prioritizing material possessions and financial resources as a measure of success.	
	EV3	Authority: Possessing the legitimate right to lead, direct, or command within a structure or organization.	
Vision	VI1	Demonstrates a clear understanding of the organization’s strategic direction.	Rafferty and Griffin (2004)
	VI2	Possesses a well-defined vision for where the unit should be in the next 5 years.	
	VI3	Lacks clarity regarding the future trajectory of the organization.	
Inspirational communication	IC1	Communicates in ways that instill pride among employees about being part of the organization.	Rafferty and Griffin (2004)
	IC2	Consistently highlights the positive aspects of the work unit.	
	IC3	Motivates individuals to view changing environments as opportunities for growth and innovation.	
Intellectual stimulation	IS1	Encourages me to approach longstanding problems with innovative perspectives.	Rafferty and Griffin (2004)
	IS2	Introduces ideas that compel me to reevaluate assumptions I had previously taken for granted.	
	IS3	Challenges me to reconsider fundamental beliefs about my work.	
Supportive leadership	SL1	Demonstrates consideration for my personal feelings before acting.	Rafferty and Griffin (2004)
	SL2	Acts thoughtfully and is attentive to my individual needs.	
	SL3	Ensures that employee interests are given appropriate priority and consideration.	
Personal recognition	PR1	Provides commendation when I perform above average.	Rafferty and Griffin (2004)
	PR2	Recognizes and acknowledges improvements in the quality of my work.	
	PR3	Personally offers compliments when I deliver exceptional performance.	
Affective organizational commitment	AOC1	I would be highly satisfied to dedicate the remainder of my career to this organization.	Park et al. (2022)
	AOC2	I take pleasure in sharing positive discussions about my organization with individuals outside of it.	
	AOC3	I genuinely feel a deep sense of ownership and responsibility toward the challenges faced by this organization.	
Satisfaction with management Satisfaction with management.	SWM1	This organization demonstrates effective and appropriate management practices.	Semedo et al. (2017)
	SWM2	I have confidence in the fairness and integrity of the organization’s management.	
	SWM3	I am satisfied with the administration of our formal recognition programs.	
	SWM4	The management adopts a progressive and forward-thinking approach.	
	SWM5	This organization operates with efficiency and organizational coherence.	
Anxiety–comfort	AC1	Experiencing heightened levels of anxiety	Semedo et al. (2017)
	AC2	Feeling a sense of concern or apprehension	
	AC3	Exhibiting signs of heightened tension or nervousness	
Depression–pleasure	DP1	Experiencing symptoms of depression	Semedo et al. (2017)
	DP2	Exhibiting feelings of happiness	
	DP3	Displaying a cheerful demeanor	
Boredom–enthusiasm	BE1	Demonstrating enthusiasm and eagerness	Semedo et al. (2017)
	BE2	Highly motivated and goal-oriented	
	BE3	Maintaining an optimistic outlook	
Tiredness–vigor	TV1	Engaging in active participation or behavior	Semedo et al. (2017)
	TV2	Remaining alert and attentive	
	TV3	Full of vitality and energy	
Anger–placidity	AP1	Displaying assertiveness or aggressive tendencies	Semedo et al. (2017)
	AP2	Experiencing anger or frustration	
	AP3	Showing signs of annoyance or irritation	

To improve the quality and credibility of the data, the questionnaire set up a concentration question, requiring respondents to select “item 5 (completely agree)” in the specified question. Questionnaires that did not select “item 5” as required would be considered invalid questionnaires. This design is intended to detect whether the respondents read and answered the questionnaire content carefully to avoid data bias caused by random answers. The content of the final version of the questionnaire and its source are shown in Table 1.

### 3.2 Data collection

The data collection subjects for this study are employees of service industry operating companies and manufacturing companies. As research objects, service industry operating companies and manufacturing enterprises are highly representative of the industry and can reveal the wide-ranging impact of leadership on employee happiness in different work environments (Wen et al., 2022). To ensure the representativeness and breadth of the data, we contacted several companies in Guangdong Province, China, and invited them to participate in the study. In the process of communicating with the company, the research team explained the purpose of the study in detail and promised to the company and employees that the data information provided by all participants would be strictly confidential to ensure the ethical compliance of the study and data privacy protection. After obtaining the consent of the company and employees, we sent the questionnaire link to the person in charge of the company by email, and the person in charge distributed it to the employees to fill in. To ensure the autonomy of participation, the questionnaire clearly informed the respondents at the beginning that this survey is completely voluntary, no personal identification information is required, and the survey data will only be used for academic research and will not involve commercial purposes.

The questionnaire survey started on August 16, 2024, lasted for 10 days and ended on August 26. A total of 600 questionnaires were distributed in this study. To ensure the quality and credibility of the data, attention questions were set in the questionnaire, and questionnaires with continuous answers or abnormal answers were eliminated. Finally, 448 valid questionnaires were collected, and the effective recovery rate reached 75%. This high recovery rate shows that the respondents have a high degree of recognition of the research, and the strict quality control measures taken by the research team during the data collection process ensure the validity and reliability of the questionnaire data. The statistical results of the questionnaire are shown in Table 2, which shows the basic information of the respondents and the distribution characteristics of the questionnaire in detail.

**Table 2:** Basic information of respondents.

Construct	Type	Quantity	Percentage
Gender	Male	201	44.87%
	Female	247	55.13%
Age	18–30	209	44.65%
	31–40	200	44.64%
	41–50	24	5.36%
	>50	15	3.35%
Education	High school and below	15	3.35%
	Associate degree	21	4.69%
	Bachelor’s degree	299	66.74%
	Master’s degree	112	25.00%
	doctoral degree	1	0.22%
Income	<5,000	69	15.40%
	5,000–9,999	160	35.71%
	10,000–14,999	95	21.21%
	15,000–19,999	85	18.97%
	>20,000	39	8.71%

In terms of gender distribution of respondents, males accounted for 44.87% and females accounted for 55.13%. The ratio of males and females was relatively balanced, which can better reflect the differences in employee happiness between different genders. In terms of age distribution, respondents aged 18–30 and 31–40 accounted for more than 80%, indicating that the respondents in this survey were mainly concentrated in the middle-aged and young groups. This group is usually the core work force of the company and may face greater work pressure and responsibilities, so it has important reference significance for studying employee happiness.

In terms of education level, respondents with bachelor’s degrees or above accounted for more than 60%, indicating that the overall education level of the respondents was high, which may reflect the target company’s high requirements for employee knowledge and skills. In terms of income distribution, respondents with monthly incomes of 5,000–9,999 yuan and 10,000–14,999 yuan accounted for more than 50%, indicating that most respondents were at a middle-income level. The happiness of this income group may be affected by many factors such as career development and work environment.

Overall, the sample structure of this study is representative, covering a variety of factors such as gender, age, education level and income level, providing a basis for studying the relationship between leadership values and employee happiness.

## 4 Result analysis

### 4.1 Confirmatory factor analysis

The study used SPSS (version 28.0) for correlation analysis and Amos (version 26.0) for confirmatory factor analysis (CFA). CFA was conducted to verify the construct of all variables and assess measurement model fit. Reliability was tested using composite reliability (Burns and Crisp), and validity was evaluated through average variance extracted (AVE) and discriminant validity. Finally, structural equation modeling was used to examine the hypothesized relationships and the overall model’s adaptability (Hair et al., 2009).

The analysis results are presented in Table 1. The overall fit index of the measurement model performed well (*χ*^2^/df = 2.233, TLI = 0.951, CFI = 0.955, RMSEA = 0.053, and SRMR = 0.051). The Cronbach’s alpha values for all scales exceeded 0.7, and the composite reliability (Burns and Crisp) reached or exceeded 0.7, confirming a high level of internal consistency within the scales (Hu and Bentler, 1999). In addition, the data demonstrated convergent validity, as the average variance extracted (AVE) for each construct exceeded 0.5, indicating that the latent variables effectively captured the variance of their respective measurement items (Hair et al., 2019). These results validate the reliability and validity of the measurement model, ensuring that the constructs are both consistent and accurate. This robust foundation provides confidence for proceeding with the structural equation model analysis to test the hypothesized relationships (Table 3).

**Table 3:** Confirmatory factor analysis.

Construct	Code	Estimate	AVE	CR	Cronbach’s alpha
Altruistic values	AV1	0.932	0.768	0.929	0.925
	AV2	0.918			
	AV3	0.927			
	AV4	0.707			
Egoistic values	EV1	0.855	0.699	0.874	0.772
	EV2	0.775			
	EV3	0.875			
Leadership	VI	0.966	0.930	0.979	0.956
	IC	0.975			
	IS	0.915			
	SL	0.920			
	PR	0.974			
Vision	VI1	0.899	0.766	0.908	0.906
	VI2	0.868			
	VI3	−0.858			
Inspirational communication	IC1	0.901	0.823	0.933	0.933
	IC2	0.911			
	IC3	0.910			
Intellectual stimulation	IS1	0.907	0.659	0.852	0.879
	IS2	0.748			
	IS3	0.772			
Supportive leadership	SL1	0.766	0.704	0.876	0.902
	SL2	0.805			
	SL3	0.937			
Personal recognition	PR1	0.904	0.850	0.944	0.944
	PR2	0.932			
	PR3	0.929			
Affective organizational commitment	AOC1	0.788	0.576	0.803	0.815
	AOC2	0.717			
	AOC3	0.771			
Satisfaction with management	SWM1	0.900	0.822	0.959	0.959
	SWM2	0.898			
	SWM3	0.908			
	SWM4	0.908			
	SWM5	0.920			
Happiness	AC	0.717	0.755	0.937	0.928
	DP	0.969			
	BE	0.979			
	TV	0.971			
	AP	0.648			
Anxiety–comfort	AC1	0.877	0.738	0.894	0.898
	AC2	0.832			
	AC3	0.867			
Depression–pleasure	DP1	0.775	0.786	0.916	0.909
	DP2	0.932			
	DP3	0.942			
Boredom–enthusiasm	BE1	0.915	0.831	0.936	0.929
	BE2	0.905			
	BE3	0.914			
Tiredness–vigor	TV1	0.928	0.810	0.927	0.928
	TV2	−0.834			
	TV3	0.934			
Anger–placidity	AP1	0.921	0.834	0.938	0.938
	AP2	0.912			
	AP3	0.906			

Overall fit index of the measurement model: *χ*^2^/df = 2.233, TLI = 0.951, CFI = 0.955, RMSEA = 0.053, SRMR = 0.052.

It is worth noting that both leadership and employee happiness were modeled as higher-order constructs to capture their multidimensional nature in this model. Leadership was conceptualized as a second-order construct comprising five first-order dimensions, including vision, inspirational communication, intellectual stimulation, supportive leadership, and personal recognition, which follows the framework proposed by Rafferty and Griffin (2004). Similarly, employee happiness was modeled as a second-order construct with five first-order dimensions, adopted from Semedo et al. (2017), representing both emotional and psychological components of employees’ well-being.

In the second-order measurement model, the fit indexes of leadership were *χ*^2^/df = 2.62, TLI = 0.978, CFI = 0.984, RMSEA = 0.060 and SRMR = 0.039; the fit indexes of employee happiness were *χ*^2^/df = 2.955, NFI = 0.971, RFI = 0.96, TLI = 0.981, CFI = 0.981, RMSEA = 0.066, and SRMR = 0.037. As highlighted by Semedo et al. (2017), these results further confirm the robust alignment between the model and the data.

To justify the aggregation of these multidimensional constructs, second-order CFA were conducted for both leadership and employee happiness. The results demonstrated that all first-order factor loadings were statistically significant and exceeded 0.70, indicating strong relationships between subdimensions and their respective higher-order constructs. Reliability and convergent validity were satisfactory: for leadership, the CR values ranged from 0.876 to 0.944 and the AVE values ranged from 0.659 to 0.850; for employee happiness, CR values ranged from 0.894 to 0.938 and AVE values from 0.738 to 0.834, all surpassing recommended thresholds. Moreover, the second-order loadings of the five dimensions on their respective constructs were consistently high, confirming that the subdimensions coherently represent their broader theoretical domains.

Therefore, consistent with theoretical precedent and empirical evidence, both constructs were treated as unified second-order latent variables in the subsequent structural equation modeling analysis.

The results of the correlation analysis are shown in Table 4, which verified the research hypothesis and showed that there is a significant relationship between the variables. The square root of AVE of each variable is higher than the correlation coefficient between it and other variables (Fornell and Larcker, 1981), which further proves that the data have good discriminant validity.

**Table 4:** Discriminant validity test results.

Constructs	AV	EV	LE	AOC	SWM	HA
AV	0.768^a^	0.054^c^	0.500	0.233	0.367	0.317
EV	0.234^b^	0.699	0.170	0.080	0.126	0.010
LE	0.707	0.412	0.930	0.437	0.532	0.472
AOC	0.483	0.283	0.661	0.576	0.515	0.517
SWM	0.606	0.355	0.730	0.718	0.830	0.564
HA	0.563	0.100	0.687	0.719	0.751	0.755

^a^Average variance extracted values are along the main diagonal.

^b^Correlations between constructs are below the main diagonal.

^c^Squared correlations between constructs are above the main diagonal.

To further address potential concerns of common method bias resulting from the use of self-reported data, we performed an additional diagnostic check. The results of Harman’s single-factor test indicated that no single factor accounted for the majority of the variance, and the one-factor model exhibited a substantially poorer fit than the measurement model (*χ*^2^/df = 9.581, CFI = 0.551, TLI = 0.539, SRMR = 0.129, RMSEA = 0.141). These results suggest that common method variance is unlikely to be a serious issue in this study.

### 4.2 Path analysis

Table 5 shows the results of path analysis. The overall fit index of the structural model performed well (*χ*^2^/df = 2.363, TFI = 0.946, CFI = 0.955, RMSEA = 0.055 and SRMR = 0.055), showing that the structural model fits the data well. The results of path analysis show that altruistic values have a significant positive impact on leadership (coefficient = 0.887, *p* < 0.001). Therefore, hypothesis 1 is supported. It shows that leaders with altruistic values can significantly improve leadership effectiveness by caring for others and focusing on teamwork. This behavior can enhance employees’ trust in leaders and stimulate employees’ work enthusiasm, thereby supporting the improvement of team and organizational performance. In contrast, Egoistic values failed to have a significant impact on leadership (coefficient = 0.018, *p* = 0.545). Therefore, hypothesis 2 is not supported. It shows that a leadership style that focuses too much on personal interests may make it difficult to play an effective role in team collaboration and employee motivation, limiting the leader’s overall effectiveness. The research results show that organizations should pay more attention to the shaping of altruistic values when cultivating leaders to meet the team’s needs for cooperation, trust, and fairness.

**Table 5:** Results of path analysis.

Path	Estimate	S. E.	C. R.	*p*-value	Result
AV-LEA	0.887	0.032	23.131	***	Supported
EV-LEA	0.018	0.031	0.606	0.545	Not supported
LEA-AOC	0.863	0.043	22.304	***	Supported
LEA-SWM	0.931	0.038	25.911	***	Supported
SWM-EH	0.689	0.039	12.226	***	Supported
AOC-EH	0.31	0.03	6.898	***	Supported

*p < 0.05, **p < 0.01, ***p < 0.001.

Leadership impacts employee happiness through two mediating paths: affective organizational commitment and manager satisfaction. Leadership has an important impact on affective organizational commitment (coefficient = 0.863, *p* < 0.001) and manager satisfaction (coefficient = 0.931, *p* < 0.001) have significant positive effects. Therefore, hypotheses 3 and 4 are supported. It shows that excellent leaders can enhance employees’ emotional attachment to the organization and increase employees’ recognition of management methods, thereby improving employee happiness (Priyanto, 2023).

Affective organizational commitment (coefficient = 0.310, *p* < 0.001) and management satisfaction (coefficient = 0.689, *p* < 0.001) have a positive impact on employee happiness. Therefore, Hypotheses 5 and 6 are supported. This shows that employees have a positive evaluation of the organization and management at both the emotional and rational levels. Affective organizational commitment mainly reflects employees’ emotional attachment to the organization, which can enhance employees’ sense of identity and belonging to the organization. When employees feel the care of the organization and the support of the leadership, they are more likely to have a sense of psychological security and belonging, which helps to enhance employees’ positive emotions and enhance their enthusiasm and loyalty to work.

### 4.3 Mediating effect

To further validate the mediating effects of affective organizational commitment and satisfaction with management in the relationship between leadership and employee happiness, a bootstrapping procedure with 5,000 resamples was performed using bias-corrected and percentile confidence intervals. The results, as shown in Table 6, revealed that both indirect pathways were statistically significant. Specifically, the indirect effect of leadership on employee happiness through affective organizational commitment was 0.345 (SE = 0.076, 95% CI [0.209, 0.507]), while the indirect effect through satisfaction with management was 0.699 (SE = 0.079, 95% CI [0.546, 0.864]). As both confidence intervals excluded zero, the mediating effects were confirmed.

**Table 6:** Result of bootstrapping analysis.

Path	Indirect effect	SE	Bias-corrected 95% CI		Percentile 95% CI	
			Lower	Upper	Lower	Upper
Indirect path						
LEA to AOC to EH	0.345	0.076	0.209	0.507	0.208	0.507
LEA to SWM to EH	0.699	0.079	0.546	0.864	0.539	0.857
Direct path						
LEA to EH	0.148	0.001	−0.342	−0.003	−0.321	0.009

In contrast, the direct effect of leadership on employee happiness was found to be non-significant (*β* = 0.148, SE = 0.001, 95% CI [−0.342, 0.009]), indicating that the relationship between leadership and employee happiness operates entirely through the mediating mechanisms of affective organizational commitment and satisfaction with management. This result provides strong empirical support for a full mediation model, suggesting that leadership enhances employee happiness not directly, but through fostering emotional attachment to the organization and improving employees’ satisfaction with managerial support.

## 5 Discussion and conclusion

### 5.1 Key findings

This study explores the impact of leadership on employee happiness from the perspective of personal values, focusing on the mechanism of altruistic values and egoistic values on leadership. By examining the mediating roles of affective organizational commitment and satisfaction with management, this study further reveals how leaders’ personal values indirectly enhance employee happiness. The results highlight the critical interplay between values, leadership, and employee happiness, showing that affective organizational commitment and satisfaction with management serve as essential pathways connecting leaders’ values to employees’ happiness.

First, leaders with altruistic values exhibit a more profound impact on leadership effectiveness compared to those with egoistic values. Altruistic values emphasize caring for others, fostering collaboration, and promoting teamwork, making leaders more likely to focus on employee needs and provide meaningful support and motivation. This, in turn, enhances employees’ trust, belonging, and commitment to the organization (Ahmad et al., 2024). While the present study found that altruistic values positively influence leadership effectiveness, suggesting their essential role in fostering trust and support, it is worth noting that the effect of altruism may not always be uniformly positive. In highly demanding or resource-constrained environments, excessive altruistic behavior might lead to leader overcommitment, emotional exhaustion, or role overload (Wang R. et al., 2024; Emmerik et al., 2005).

Unexpectedly, this study found that egoistic values have a non-significant effect on perceived leadership. The non-significant relationship may result from different contextual. In performance-oriented organizational cultures, moderate self-interest and goal-directed behaviors may be perceived as professional competence rather than selfishness, thereby neutralizing the potential negative impact of egoistic values on perceived leadership effectiveness. Another possible explanation lies in a compensatory mechanism, where egoistic tendencies are balanced by competence or achievement orientation. Employees may tolerate self-focused behaviors if leaders demonstrate strong task competence and deliver organizational results. In addition, in the Chinese context, employees may be less likely to perceive self-interested leadership behaviors as effective due to cultural expectations emphasizing harmony, empathy, and relational trust. This contextual interplay may have neutralized the overall relationship, resulting in a statistically non-significant effect.

Furthermore, this study highlights that the dual effects of leadership on employees’ emotional (affective organizational commitment) and cognitive (satisfaction with management) levels ultimately translate into significant improvements in overall employee happiness. The perceived leadership exerts an indirect positive influence on employee happiness through affective organizational commitment and satisfaction with management. Affective organizational commitment originates at the emotional level, reflecting employees’ attachment, identification, and sense of belonging to the organization (Pulido-Martos et al., 2024). This emotional connection fosters higher levels of psychological happiness, work enthusiasm, and resilience in employees. On the other hand, satisfaction with management reflects employees’ subjective evaluations of their managers’ behaviors, communication styles, and supportiveness (Vereb et al., 2024). Positive satisfaction with management contributes to a favorable perception of the work environment, reducing stress and enhancing workplace harmony (Qi et al., 2023). These two dimensions demonstrate how leadership behaviors rooted in personal values can simultaneously strengthen emotional and cognitive aspects of employee happiness. This dual pathway provides robust theoretical support for understanding the mechanisms through which leadership influences employees’ happiness.

In conclusion, this study demonstrates that leaders’ personal values play a pivotal role in shaping employees’ psychological state and job satisfaction. The findings provide organizations with actionable insights to optimize leadership development by embedding value-driven strategies into their leadership programs. Specifically, organizations should prioritize fostering altruistic values among leaders to create a supportive, inclusive, and collaborative workplace culture. At the same time, organizations should encourage leaders to balance performance goals with employee needs to achieve sustainable organizational success. This study contributes a novel theoretical perspective by elucidating the mechanisms of leadership influence on employee happiness and offers practical guidance for improving leadership effectiveness in modern organizations.

### 5.2 Theoretical contributions

This study has important theoretical and practical significance for improving employee happiness in organizations. By exploring how leaders’ egoistic values and altruistic values affect employee happiness through leadership, this study provides more precise guidance for organizations to formulate leadership development plans and value cultivation strategies.

From a theoretical perspective, this study enriches the research framework between leadership and employee happiness and further explains the key role of leaders’ values in influencing employee happiness. With the personal values of leaders as the main research focus, this study expands its application scope in the field of organizational behavior. This study emphasizes the important role of altruistic values in shaping positive leadership behaviors and their impact on employee happiness, expands the research on leadership’s impact on employee happiness from the perspective of values, and provides a new theoretical perspective for organizational behavior research.

Compared with previous studies that focus on the impact of work content diversity (Hafeez et al., 2024) and leadership management ability (Kaur and Kaur, 2024) on employee happiness, this study deeply explores the role of leaders’ values on employee happiness from an emotional and rational level, providing a theoretical basis for future research on the impact of other values on employee happiness. This study deeply explored the profound impact of leaders on employee happiness through the emotional level and the rational level, revealing the internal mechanism of this process. This lays a theoretical foundation for subsequent research to explore the role of other values on employee happiness. This study found that altruistic values can improve happiness by enhancing employees’ emotional belonging and identification with the organization, and this improvement is mainly manifested in employees’ positive emotional attitudes toward the organization and higher job satisfaction. In addition, leaders with altruistic values are likely to create a working atmosphere with unity and cooperation as the core, further strengthening employees’ happiness and loyalty to the organization. On the contrary, leaders with egoistic values focus on personal achievement and maximization of interests, which leads to employees feeling a lack of emotional support and reduced team trust in the organization, which has no impact on leadership. This finding provides guidance for organizations in developing leaders on how to balance the values of egoistic and altruism and maintain sustainable improvements in employee happiness.

### 5.3 Practical contributions

From a practical perspective, the results of this study show that leaders with altruistic values are better able to enhance employee happiness through positive leadership behaviors. Leaders with altruistic values tend to display caring and supportive behaviors, which can effectively promote employees’ emotional belonging and identification with the organization, thereby creating a collaborative and trusting work environment. Therefore, in the process of organizational practices, organizations should pay more attention to shaping and strengthening altruistic values. First, organizations can integrate altruistic value orientation into leadership selection and training processes. During recruitment and promotion, personality assessments (e.g., the Schwartz Value Survey) and situational judgment tests can be employed to evaluate candidates’ altruistic tendencies, empathy, and team orientation.

Second, organizations should establish performance evaluation systems that emphasize supportive leadership. Traditional appraisal systems often prioritize financial or performance outcomes, which may inadvertently overlook the relational aspects of leadership. To encourage altruistic leadership, organizations can include process- and relationship-oriented indicators—such as supporting employee growth, promoting team collaboration, and cultivating a culture of well-being. Employee feedback systems could also include items like “My supervisor cares about my well-being,” reinforcing altruistic behaviors through long-term performance incentives.

Moreover, organizations can strengthen an altruism-oriented culture through symbolic and institutional mechanisms. This may include recognition programs such as “Altruistic Leadership Awards” or “Empathetic Manager of the Year,” as well as embedding people-centered principles into corporate mission statements and managerial codes of conduct. These practices can transform altruism from an individual virtue into a shared organizational norm, fostering a more supportive and collaborative environment that enhances both employee happiness and organizational performance.

In summary, this study reveals the important impact of leader values on employee happiness from both theoretical and practical perspectives, and further clarifies that leadership promotes employee happiness at the emotional level (effective organizational commitment) and rational level (satisfaction with management). The results highlight the key role of altruistic values in shaping positive leadership behaviors, improving employee happiness, and promoting organizational performance. At the same time, this study also reveals the limitations of egoistic values in leadership development and the negative impact they may have on team trust building and employee happiness. Through this study, organizations can better understand how to improve employee happiness by shaping leader values, thereby achieving sustainable development of individual, team, and organizational goals.

### 5.4 Limitations and future research

This study explored the impact of leadership on employee happiness from the perspective of values, which is innovative but also has some limitations.

First, this study mainly focused on two personal values, egoism and altruism, but did not explore other values to maintain theoretical clarity and model parsimony. Future research could extend this framework by incorporating additional cultural or personality-based values, such as collectivism or openness, to provide a more comprehensive understanding of how diverse value systems shape perceived leadership and employee happiness across different organizational and cultural contexts. Additionally, job characteristics may also act as potential moderating factors, as leadership effectiveness and employee happiness are often influenced by the nature of work, such as task interdependence, job autonomy. Future research is therefore encouraged to extend this framework by integrating job characteristics or other contextual moderators to identify the boundary conditions under which leadership values exert stronger or weaker effects on employee outcomes.

Second, this study used a questionnaire to collect data. Despite several methodological remedies adopted to minimize bias, including strict respondent screening, confirmatory factor analysis for construct validity, and a statistical test for common method variance, the use of a self-reported questionnaire may still involve subjective evaluations and socially desirable responding. Future research can use mixed methods, combining qualitative research and quantitative analysis, to deeply explore the key components of leadership and its internal mechanisms, to improve the comprehensiveness of the research results. Besides, future research could incorporate objective performance indicators to validate and extend the present findings from multiple perspectives.

Third, it should be noted that the non-significant effect of egoistic values does not necessarily imply that such values are entirely ineffective. Egoistic orientations may yield positive outcomes under specific boundary conditions characterized by strong competition or high-performance pressure. Conversely, excessive altruism may generate unintended negative consequences, such as emotional exhaustion or diminished decision-making efficiency. Therefore, future research could further investigate the dual effects of altruism and self-interest to develop a more comprehensive and nuanced understanding of how these value orientations shape perceived leadership effectiveness across different organizational contexts.

Finally, the data of this study are mainly based on employees’ self-evaluation, which may be affected by the subjective perception of the respondents. For example, employees’ evaluation of leaders may be related to their personal experience, emotional state, or role expectations, which may lead to subjective bias in some conclusions. To overcome this limitation, future research can introduce multiple data sources and collect more objective data by comparing employee evaluations with leaders’ self-evaluations. In addition, more control variables can be introduced, such as organizational size, industry characteristics, and the nature of employee positions, to more comprehensively reveal the mechanism by which leadership affects employee happiness.

In summary, although this study provides important theoretical and practical contributions in exploring the relationship between leadership and employee happiness. Future research can be improved in terms of the diversity of values, the diversity of methods, and the objectivity of data to further deepen the understanding of leadership and its impact.

## Data Availability

Data supporting this article will be made available upon request to the corresponding author.
