# Correction: How culture values shape leadership and employee well-being: insights from altruistic and egoistic perspectives

**DOI:** 10.3389/fpsyg.2026.1788659

**Published:** 2026-01-28

**Authors:** Zhirong Tian, Qian Wen, Haoming Ding, Yuan Zhu

**Affiliations:** 1 School of Economics and Management, Guangxi University of Science and Technology, Liuzhou, Guangxi, China; 2 Hoseo University, Asan-si, Republic of Korea; 3 School of Humanities, Arts and Design, Guangxi University of Science and Technology, Liuzhou, Guangxi, China

**Keywords:** leadership, altruistic values, egoistic values, employee happiness, affective organizational commitment, satisfaction with management

Author [Qian Wen] was erroneously spelled as [Wen Qian Wen].

The original version of this article has been updated.

